# Comparative study of endometrioid borderline ovarian tumor with and without endometriosis

**DOI:** 10.1186/s13048-018-0440-x

**Published:** 2018-08-11

**Authors:** Wen Zhang, Shuangzheng Jia, Yang Xiang, Junjun Yang, Congwei Jia, Jinhua Leng

**Affiliations:** 1 Department of Obstetrics and Gynecology, Peking Union Medical College Hospital, Chinese Academy of Medical Sciences and Peking Union Medical College, No. 1 Shuaifuyuan, Dongcheng District, Beijing, 100730 China; 2 Department of Pathology, Peking Union Medical College Hospital, Chinese Academy of Medical Sciences and Peking Union Medical College, No. 1 Shuaifuyuan, Dongcheng District, Beijing, 100730 China

**Keywords:** Endometrioid borderline ovarian tumor, Endometriosis-associated endometrioid borderline ovarian tumor, Endometriosis

## Abstract

**Background:**

Synchronous endometriosis has been poorly studied in women with endometrioid borderline ovarian tumors (EBOTs). The aims of this study were to compare the clinicopathological features and prognosis of EBOTs with or without endometriosis.

**Results:**

Of 52 patients diagnosed with EBOTs, no death was observed and only one case had successful pregnancy during the follow-up period. Older, menopausal EBOT patients, EBOT patients with small tumors and relatively low CA125 level probably had better progression-free survival (PFS) outcomes. About 1/3 of EBOTs had concomitant endometrial lesions. Approximately 1/3 of EBOTs were associated with endometriosis. Patients were divided into two groups according to the presence or not of endometriosis in this retrospective cohort study. Patients with endometriosis-associated endometrioid borderline ovarian tumor (EAEBOT) were more likely to be younger and premenopausal. Variables such as PFS outcomes, endometrial lesions did not differ statistically between groups. However, in specific EBOT patients like parous patients, patients with CA125 ≥ 140 U/ml or patients without fertility sparing surgery, coexisting endometriosis perhaps predicted worse PFS outcomes.

**Conclusion:**

We considered EAEBOT as an entity similar to non-EAEBOT. Closely follow-up for some particular patients with concomitant endometriosis was necessary.

## Background

Endometrioid ovarian epithelial tumors occur as endometrioid cystadenomas, endometrioid cystadenofibromas, endometrioid borderline ovarian tumors (EBOT) and endometrioid carcinomas [1]. Thereinto, endometrioid borderline ovarian tumor, second to the serous and mucinous borderline ovarian tumor (BOT), is characterized with atypical or histologically malignant endometrioid type glands or cysts without stromal invasion in accordance with WHO criteria [2].

Endometriosis is a frequent gynecological disease, which has been evaluated that 0.5–1% of the endometriosis patients are associated with neoplasia [3]. The most common endometriosis-associated malignant ovarian tumors are endometrioid carcinomas and clear cell carcinomas [4]. Endometriosis–associated borderline ovarian tumors are less common when compared with the endometriosis-associated malignant ovarian tumors [5]. Previous endometriosis history or discovery of endometriosis during the histological analysis is frequent in EBOTs [2]. Concomitant endometriosis was identified in 12 patients from 33 EBOTs in one research [6] and 16 out of 31 EBOTs had ovarian or ovarian and/or vaginal endometriosis in another research [7] which raised the major question of relationship between EBOT and endometriosis. Previous studies had demonstrated no associations between endometriosis and the prognosis of ovarian endometrioid carcinomas and ovarian clear cell carcinomas [8, 9]. However, no studies had compared endometriosis-associated EBOT (EAEBOT) with non-EAEBOT in terms of clinical and pathological features and prognosis. Therefore, in the present study, we aimed to identify whether EAEBOT represented a heterogeneous disease distinct from other forms of EBOTs in aspects of clinical, pathological features and prognosis.

## Methods

After obtaining Institutional Review Board approval for medical record review, patients with a primary histopathology diagnosis of borderline ovarian tumor of endometrioid histotype at the Peking Union Medical College Hospital were identified and included in this retrospective cohort study from 1995 to 2015. Subjects with malignant ovarian tumors or borderline ovarian tumors of other histotypes were excluded.

All patients were treated in our institution and followed up in outpatient department or by telephone. No patients had chemotherapy before the surgery. Patients had undergone either a laparotomic or a laparoscopic approach. Staging surgical procedures had been performed dependent on the surgical teams, on whether the EBOT had been diagnosed during or after the surgical procedure and on disease extension. The International Federation of Gynecology and Obstetrics (FIGO) 2013 staging system for epithelial ovarian tumors was used for determining disease stage based on the operative descriptions and pathology records [10]. Staging surgery was performed wherein all peritoneal surfaces were carefully inspected using peritoneal washing, random or oriented multiple biopsies, and infracolic omentectomy [11]. Conservative surgery was defined as fertility sparing wherein the uterus and at least part of one ovary are salvaged, whereas radical surgery was defined as bilateral salpingo-oophorectomy with or without a hysterectomy. Microscopic slides were reviewed and confirmed by two experienced gynecologic pathologists.

**Fig. 1:**
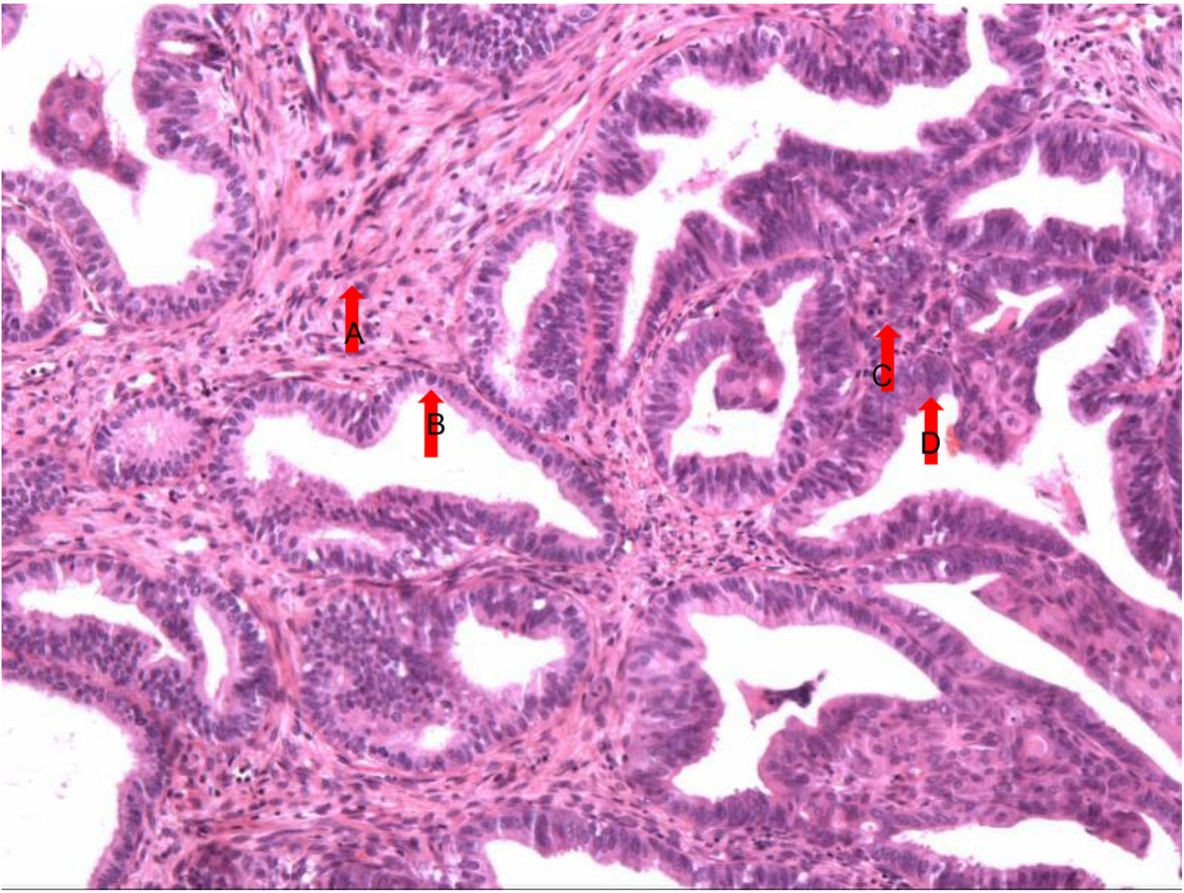
Endometrioid borderline ovarian tumors (EBOT) and adjacent endometriotic lesions. Caption: **a** normal endometrial stroma; **b** normal endometrial glandular epithelia; **c** EBOT stroma; **d** EBOT glandular epithelia.

Patients were divided into two groups according to the detection of EBOT arising from endometriosis or not. Specifically, EAEBOT was defined as follows: [1] presence of EBOT and endometriosis in the same ovary (see Fig. 1), [2] presence of endometriosis in one ovary and of EBOT in the contralateral ovary, [3] presence of EBOT and extraovarian endometriosis. Figure 1 shows the concomitant endometriosis and EBOT in the same ovary.

Medical records were comprehensively reviewed, and variables such as patient’s age, fertility status, gravidity number, symptoms, menopausal status, preoperative serum CA-125 levels, surgery date, maximal tumor diameter, cyst rupture, FIGO stage, laterality of tumor, surgical approach (laparoscopy or laparotomy), whether comprehensive staging surgery or fertility sparing surgery was performed, chemotherapy, concomitant endometriosis, date of disease progression or recurrence were collected. The simultaneous detection of endometrial cancer (EC) or endometrial intraepithelial neoplasia (EIN) in the surgical specimen was also reported, and in cases without hysterectomy uterine curettage was performed after surgery to obtain endometrial pathology. Normal upper limit of serum CA 125 was 35 U/ml and we also set another CA125 indicator of which upper limit was 140 U/ml (four times the normal value). Progression-free survival (PFS) was defined as the time interval from the date of primary surgery to the date of disease progression or recurrence.

Statistical analyses were performed using SPSS version 20.0 (SPSS Inc.). Continuous variables were analyzed by t-test and categorical variables were analyzed by Chi square test or Fisher exact test to assess the significance of differences in clinical and pathological features between EAEBOTs and non-EAEBOTs. Survival analysis was obtained using the log-rank test in Kaplan–Meier method. Variables with statistical significance in univariate analyses were included in the multivariate analysis. Multivariate analysis was performed using the Cox proportional hazards regression model to identify predictors of survival. All *P* values reported were two tailed, and *P* < 0.05 was considered statistically significant.

## Results

During the study period, a total of 52 patients met the inclusion criteria. Of them, 19(36.5%) patients were associated with endometriosis and allocated to group 1, while the other 33(63.5%) without endometriosis were assigned to group 2. Mean age ± SD at diagnosis of the entire cohort was 41.9 ± 11.6 (range 23-81 years) years old. Twelve (23.1%) were in menopause. Fifteen (29.4%) had at least 3 gravidities and 24(46.2%) were nulliparous. Distribution based on FIGO stage was 49(94.2%) for stage I, 2 for stage II, 1 for stage III, and 0 for stage IV. Forty-seven (90.4%) patients were with unilateral tumors. Twelve (23.1%) patients received chemotherapy after primary surgery. Of 52 cases, 8(15.4%) had concomitant EIN and 11(21.2%) had EC.

The clinical and pathological variables between groups are shown in Table 1. Patients with endometriosis were younger than those without endometriosis, which was statistically significant (*P* = 0.040). Consistent with age, none of the patients in group 1 and 36.4% in the group 2 were menopausal (*P* = 0.002). There was no difference between these two groups in terms of EC (*P* = 0.503) or EIN (*p* = 0.694). The most common symptom for both groups was pelvic mass, followed by vaginal bleeding. Symptoms did not differ statistically between the two groups. There were also no differences between two groups in terms of CA125 level, tumor size, FIGO stage and symptom.

**Table 1:** Clinical and pathological characteristics of the patients

Characteristics	EAEBOT (*n* = 19)	Non-EAEBOT (*n* = 33)	*P*
Age(years)(mean ± SD)	39.9 ± 7.5	43.1 ± 13.3	0.280
Age ≥ 50 years	1(5.3%)	10(30.3%)	0.040
Nulliparous	9(47.4%)	15(45.5%)	0.894
Gravidity number > 2	4(21.1%)	11(33.3%)	0.405
Symptom			
Pelvic mass	19(100.0%)	28(84.8%)	0.145
Vaginal bleeding	2(10.5%)	7(21.2%)	0.458
Pain	1(5.3%)	5(15.2%)	0.397
Torsion	0(0.0%)	2(6.1%)	0.527
Distension	1(5.3%)	0(0.0%)	0.365
Menopause	0(0.0%)	12(36.4%)	0.002
CA125 > 35 U/ml	15(78.9%)	18(54.5%)	0.091
CA125 ≥ 140 U/ml	7(36.8%)	8(24.2%)	0.376
Maximal tumor diameter ≥ 10 cm	3(15.8%)	7(21.2%)	1.000
Cyst rupture	14(73.7%)	24(72.7%)	1.000
FIGO stage			1.000
I	18(94.7%)	31(93.9%)	
II,III,IV	1(5.3%)	2(6.1%)	
Tumor side			0.145
Unilateral	19(100.0%)	28(84.8%)	
Bilateral	0(0.0%)	5(15.2%)	
Endometrial pathology			
Endometrial cancer	5(26.3%)	6(18.2%)	0.503
EIN	2(10.5%)	6(18.2%)	0.694

*EAEBOT* endometriosis-associated endometrioid borderline ovarian tumor,

*EIN* endometrial intraepithelial neoplasia

After a median follow-up time of 30 months (range 6–177 months) post treatment, 9 (17.3%) disease progressions or recurrences were observed and 43 (82.7%) were censored at last follow-up in the entire study cohort. No disease-specific deaths were observed. Two cases of malignant transformation into endometrioid ovarian cancer were observed after 18 and 68 months respectively during the follow-up, of which one is EAEBOT and the other is non-EAEBOT. Of the 25 women with fertility sparing surgery, only one woman with non-EAEBOT on stage IC had two successful term births during the follow-up time and the first birth was 1 year after the surgery. Median PFS was 169 months for the entire cohort. Table 2 shows the results of uni-variate and multivariate progression free survival analysis. In uni-variate analysis, age, menopause status, CA125 level (cutoff value = 140 U/ml) and tumor size were significant prognostic factors for PFS. Patients who were older than 50 years old, in menopause, with CA 125 < 140 U/ml, with maximal tumor diameter < 10 cm had better PFS outcomes and *P* values were 0.031, 0.023, 0.019 and 0.040, respectively. However, concomitant presence of endometriosis was not significantly associated with PFS outcomes. There were also no relationships between PFS outcomes and the following variables, like surgical approach (laparoscopy or laparotomy), whether comprehensive staging surgery was conducted, whether fertility sparing surgery was performed, cyst rupture, FIGO stage, whether patients received chemotherapy after surgery, concomitant endometrial lesion and tumor side (unilateral or bilateral). Multivariate Cox regression survival analysis has been performed in the whole cohort controlling for confounding factors. No factors were confirmed to be independent predictors of PFS.

**Table 2:** Predictors of progression-free survival in univariate and multivariate survival analysis

Characteristics		*n*	P(univariate analysis)	P(multivariate analysis)	P(stratified analysis)
Age	< 50 years	41(78.8%)	0.031	1.000	0.733
	≥50 years	11(21.2%)			–
Nulliparous	No	28(53.8%)	0.548	–	0.045
	Yes	24(46.2%)			0.315
Gravidity	≤2	36(70.6%)	0.746	–	0.492
	> 2	15(29.4%)			0.536
Menopause	No	40(76.9%)	0.023	0.983	0.834
	Yes	12(23.1%)			–
CA125	< 35 U/ml	15(31.3%)	0.893	–	0.336
	≥35 U/ml	33(68.7%)			0.103
CA125	< 140 U/ml	33(68.7%)	0.019	0.344	0.301
	≥140 U/ml	15(31.3%)			0.045
Maximal tumor diameter	< 10 cm	40(80.0%)	0.040	0.265	0.292
	≥10 cm	10(20.0%)			0.077
Surgical approach	Laparoscopy	22(42.3%)	0.890	–	0.480
	Laparotomy	30(57.7%)			0.120
Comprehensive staging surgery	No	23(44.2%)			0.824
	Yes	29(55.8%)	0.586	–	0.108
Fertility sparing surgery	No	27(51.9%)	0.697	–	0.019
	Yes	25(48.1%)			0.272
Cyst rupture	No	12(24.0%)	0.055	–	–
	Yes	38(76.0%)			0.517
FIGO stage	I	49(94.2%)	0.651	–	0.282
	II、III、IV	3(5.8%)			–
Chemotherapy after surgery	No	40(76.9%)	0.387	–	0.851
	Yes	12(23.1%)			0.441
Endometrial pathology	Without EIN/EC	33(63.5%)	0.830	–	0.122
	EIN	8(15.4%)			–
	EC	11(21.2%)			0.371
Tumor side	Unilateral	47(90.4%)	0.076	–	0.121
	Bilateral	5(9.6%)			–
Endometriosis	No	33(63.5%)	0.315	–	–
	Yes	19(36.5%)			–

*EC* endometrial cancer, *EIN* endometrial intraepithelial neoplasia

Table 2 also shows the results of the differences of PFS outcomes between two groups after stratification by other confounding factors. For parous patients, patients with CA125 ≥ 140 U/ml or patients with radical surgery, EBOT patients with coexisting endometriosis had worse PFS outcomes compared with those without endometriosis and *P* values were 0.045, 0.045 and 0.019, respectively. However, for nulliparous patients, patients with CA125 < 140 U/ml or patients with fertility sparing surgery, there were no significant differences in terms of PFS outcomes between EAEBOT patients and non-EAEBOT patients.

## Discussion

Our study showed that most patients with EBOTs were young, premenopausal and overwhelming majority of patients had stage I diseases. During the follow-up, 9 cases showed disease progressions or recurrences and only one woman had successful term birth. Older, menopausal patients and patients with relatively low CA125 (< 140 U/ml) and relatively small tumors (< 10 cm) probably had better PFS outcomes. Patients with EAEBOT were younger and more likely to be premenopausal. Variables such as FIGO stage, endometrial lesions did not differ statistically between the two groups. Concomitant Endometriosis was not associated with PFS outcomes. However, in some specific type of patients, like parous patients, patients with CA125 ≥ 140 U/ml or patients without fertility sparing surgery, coexisting endometriosis perhaps predicted worse PFS outcomes.

How ovarian endometriosis developed into ovarian neoplasm had been debated for decades from biological, epidemiological and clinical perspectives [12–16]. Several studies reported concomitant endometriosis in EBOTs [2, 6, 7, 17] which was in line with our results. A substantially increased risk of BOT or ovarian cancer was observed in endometriosis patients, additionally, hazard ratios associated with endometriosis were reported to be 12.4 for ovarian cancer and 5.5 for BOT [18]. Such data indicated the possibility of EBOT developed from endometriosis, possibly via the stage of atypical endometriosis [19]. While the pathogenesis of EAEBOT remained unclear, atypical endometriosis could be found in some endometriosis associated ovarian tumors and was considered to be a intermediate link during the neoplastic progression [5].

Present studies have shown that endometriosis-associated ovarian carcinoma (EAOC) might deviate from the non-EAOC [9]. However, as for EBOTs, EAEBOT and non-EAEBOT showed almost the same clinical and pathological features, as well as the PFS. In terms of clinical and pathological features, both groups showed no significant differences in most aspects except for age and menopausal status. Consistent with our study, previous studies also indicated that endometriosis-associated neoplasms were frequently found in younger women [5]. This was probably because of the symptoms of endometriosis like dysmenorrhea, dyspareunia, and/or pelvic mass so that these patients were more likely to see a doctor and then detected the ovarian tumors at earlier ages.

According to our results, prognoses of younger patients, premenopausal patients or patients with relatively high CA125 level or relatively large tumors were likely to be poor. Younger and premenopausal patients are still under the effect of sex hormone which could exert an effect of hormonal field effect. Field effect, also described as field cancerization, means a field of cellular and molecular aberrations, which predisposes to the initiation and progression of tumor [20]. Sex hormone in younger women could probably help constitute a field of susceptibility to endometriosis and endometrioid cell-type tumors, as well as the recurrence or progression of diseases, which also could account for the phenomenon that younger and premenopausal patients have worse prognosis. What is more, patients with relatively high CA125 level or relatively large tumors were likely to have poor prognoses. One case report shows that even a slight CA125 increase can be indicative of a poor prognosis [21], which supports our results. And as described in one article that CA125 level is positively correlated with the tumor size [22], it could be deduced from the above evidences that large tumor size is probably also an indicator of poor prognosis. Up to this point, these patients should pay attention to the recurrence of EBOT during follow-up. As for the association between endometriosis and the prognosis of EBOT, on one hand, the current published articles served to demonstrate that ovarian tumors associated with endometriosis had a better prognosis than those without endometriosis [19]. On the other hand, two articles about endometrioid ovarian cancer and ovarian clear cell carcinoma demonstrated that no significant differences in overall survival or progression-free survival could be found between patients with or without endometriosis [8, 9]. While in our study, tumors with concomitant endometriosis did not show better or worse PFS outcomes when compared with those without. Nevertheless, when compared with non-EAEBOT patients, EAEBOT patients presented with worse PFS outcomes in some particular patients (parous patients, patients with CA125 **≥** 140 U/ml or with radical surgery). The above mentioned hormonal field effect could also be used to explain the phenomenon. The specific hormonal field effect predisposes to the development of endometriosis and EBOT, and endometriosis could lead to the increase of CA125. Extremely increased CA125 level and endometriosis found in the parous patients (pregnancy is usually considered be the protective factor for endometriosis) probably indicate the severe endometriosis, which may imply the stronger hormonal field effect and the strong effect could probably increase the risk for progression or recurrence of EBOT. Furthermore, for parous patients and patients with CA125 **≥** 140 U/ml, radical surgery are often suggested for them, which could indirectly lead to results that EAEBOT patients with radical surgery may have poor prognosis. Given these findings, for EAEBOT patients with the above conditions, strict follow-up would be necessary for them in case of recurrence or disease progression.

A systematic review evaluated the fertility outcome after serous and mucinous BOT management. Conservative management of early stage BOT resulted in a spontaneous pregnancy rate of 54% and 34% in advanced stage BOT [23]. However, there was only one (4%) case of successful term birth in women with conservative surgery which demonstrated a poor fertility outcome of EBOT.

It is noteworthy that about one third of EBOTs had synchronous endometrial disorders in our study. Previous studies also reported concomitant endometrial lesions such as endometrioid adenocarcinoma [2], atypical hyperplasia [6, 7], simple hyperplasia [6, 7], polyps [7, 17, 24] in EBOTs. Concerning these evidences, uterine curettage was suggested for the patients with conservative surgery and hysterectomy was advised for patients underwent radical surgery. Our article also briefly evaluated the relationship between synchronous endometrial lesion and endometriosis. Unlike the results of previous study which reported that rate of endometrial cancer diagnosis was significantly higher in women with endometriosis associated endometrioid ovarian cancer than in the other patients [8], our study did not find the association between concomitant endometriosis and synchronous endometrial disease.

The strengths of our study include the following aspects. Firstly, in order to identify whether EAEBOT represented a separated entity distinct from the non-EAEBOT, which was never had been studied, we performed comparisons between EAEBOT and non-EAEBOT patients in aspects such as clinical and pathological features, synchronous endometrial lesion and PFS. Secondly, we described the clinical and pathological characteristics of EBOT, reported information about the treatment of EBOTs, and provided the detailed follow-up data which efficiently made up the deficiencies of current studies due to the rarity of this kind of disease. Thirdly, we also focused the synchronous endometrial disorders in EBOT and explored the association between concomitant endometriosis and endometrial disorder which,to the best of our knowledge, had never been discussed. Fourthly, we reported the reproductive outcomes after conservative surgery for EBOTs, and according to our results, we distinguished the population with worse PFS outcomes in EBOT and EAEBOT women in which close follow-up should be suggested for them.

This study has, however, some limitations. Firstly, when interpreting the results of this study, one thing must be pointed out that the sample size was not sufficient which perhaps could lead to false negative results such as the inability to discover the differences between EAEBOTs and non-EAEBOTs and identify more risk factors affecting PFS, which should be considered with caution. Study with a large sample size is needed to verify our present study results or come up with novel theory. Secondly, study from a single academic institution often involved in the selection biases. Thirdly, current findings that consider EAEBOT as an entity similar to non-EAEBOT were derived mostly from the clinical results of our study, which required confirmation at the molecular level such as gene diagnosis.

## Conclusions

There were no significant differences between EAEBOT and non-EAEBOT in many main aspects of clinicopathological features and prognosis, thus we considered EAEBOT as an entity similar to non-EAEBOT. Patients with EAEBOT were more likely to be younger and premenopausal. Close follow-up for some particular patients with endometriosis was necessary.
